# Complications Following Percutaneous Epidural Neuroplasty: A Narrative Review of Clinical Evidence and the Rationale for Post-Procedural 6 h Inpatient Monitoring Amid Limited Systematic Data

**DOI:** 10.3390/medicina61081397

**Published:** 2025-08-01

**Authors:** Jae Hun Kim, Eun Jang Yoon, Sung Ho Jo, Sun Ok Kim, Dong Woo Lee, Hwan Hee Kim

**Affiliations:** 1 Department of Anesthesiology and Pain Medicine, Konkuk University School of Medicine, Seoul 05030, Republic of Korea; painfree@kuh.ac.kr; 2 Department of Pain Medicine, Kok Pain Clinic, Seongnam-si 13615, Gyeonggi-do, Republic of Korea; eunjang.yoon@gmail.com (E.J.Y.); emily.sunoc@gmail.com (S.O.K.); 3 Department of Neurology, Kok Pain Clinic, Seongnam-si 13615, Gyeonggi-do, Republic of Korea; josungho6775@gmail.com; 4 Department of Rehabilitation Medicine, Kok Pain Clinic, Seongnam-si 13615, Gyeonggi-do, Republic of Korea; figo0115@naver.com

**Keywords:** adhesiolysis, complications, epidural neuroplasty, monitoring, physiologic, narrative review, patient safety

## Abstract

*Background*: Percutaneous epidural neuroplasty (PEN) and related adhesiolysis procedures are widely used for managing chronic spinal pain. Although generally safe, complications—ranging from minor to life-threatening—have been reported. This review aimed to estimate the incidence and characteristics of complications following PEN and to evaluate the medical rationale for post-procedural inpatient monitoring. *Methods*: We systematically searched PubMed, Embase, and the Cochrane Library for studies published from January 2000 to April 2025 reporting complications associated with PEN. We performed a random-effects meta-analysis on five eligible cohort studies to estimate the pooled complication rate and evaluated heterogeneity. Risk of bias was assessed using the Newcastle–Ottawa Scale. *Results*: Five cohort studies (n = 1740) were included in the meta-analysis, with a pooled complication rate of 9.0% (95% CI: 4.8–13.1%, I^2^ = 97.5%). A total of 133 complications were identified from cohort studies and case reports. Mechanical and neurological complications were most common. Serious complications, including hematoma, meningitis, and cardiopulmonary arrest, were concentrated within the first 6 h post-procedure. *Conclusions*: This meta-analysis highlights a quantifiable risk of complications associated with PEN. Our findings support structured inpatient monitoring during the immediate post-procedural period to enhance safety and outcomes.

## 1. Introduction

Percutaneous epidural neuroplasty (PEN), also known as epidural adhesiolysis, is an interventional pain management technique widely employed for treating chronic spinal pain conditions resistant to conventional therapies [1]. These conditions frequently include failed back surgery syndrome (FBSS), spinal stenosis, degenerative disc disease, and recurrent disc herniations [2,3]. Developed initially in the late 1980s by Dr. Gabor Racz, PEN involves inserting a steerable catheter into the epidural space under fluoroscopic or epiduroscopic guidance, allowing for targeted mechanical disruption of epidural adhesions combined with precise pharmacological administration of steroids, local anesthetics, hyaluronidase, and, occasionally, either hypertonic saline or ozone [4,5,6,7,8,9,10,11]. In this manuscript, the terms percutaneous epidural neuroplasty (PEN), adhesiolysis, and neuroplasty are used interchangeably. While some procedural nuances exist—such as the use of specific catheters or balloon devices—the overarching technique and intent are consistent across the literature. For clarity, all three terms are treated as synonymous throughout this review.

Despite its growing popularity due to its minimally invasive nature and promising therapeutic outcomes, PEN is associated with a range of complications. Documented adverse events vary broadly in severity and clinical impact, ranging from minor symptoms, such as transient paresthesia and mild positional headaches, to potentially severe and life-threatening complications, including dural puncture, epidural hematoma, meningitis, pneumocephalus, and severe cardiopulmonary reactions [12,13]. These complications are of particular concern given the vulnerable patient populations often selected for PEN instead of surgery, including elderly patients or those with altered spinal anatomy from previous surgical interventions [14,15,16,17,18,19,20,21].

To date, while numerous individual studies and case reports have documented these complications, a consolidated narrative analysis that integrates multidisciplinary clinical perspectives and systematically evaluates the comprehensive range of complications has been lacking. Existing reviews have primarily focused on procedural efficacy or provided limited analyses based on smaller patient cohorts.

This narrative, incorporating selected elements of systematic methodology, is conducted to address the current gap by synthesizing findings from clinical studies, including case reports, observational studies, randomized trials, and selected review articles. Our aim is to systematically classify and evaluate the types, frequencies, and severities of complications associated with PEN while also exploring patient-specific risk factors. Furthermore, we provide a multidisciplinary discussion supporting the medical rationale for short-term inpatient monitoring after PEN, which may improve patient safety through early detection and timely intervention.

## 2. Materials and Methods

### 2.1. Search Strategy and Selection Criteria

We conducted this meta-analysis following the Preferred Reporting Items for Systematic Reviews and Meta-Analyses (PRISMA) guidelines.

A comprehensive search was performed across PubMed, Embase, and the Cochrane Library for English-language human studies published between 1 January 2000 and 12 April 2025. Search terms included (“neuroplasty” OR “epidural lysis” OR “racz” OR “lysis of adhesion”) AND (“adverse” OR “complication” OR “complications”).

We included randomized controlled trials, observational cohort studies, and case series with ≥10 patients that reported complications explicitly related to percutaneous epidural neuroplasty (PEN). We excluded review articles, letters, editorials, animal studies, and case reports for the meta-analysis, though relevant case reports were cited narratively to illustrate rare or severe complications.

**Figure 1:**
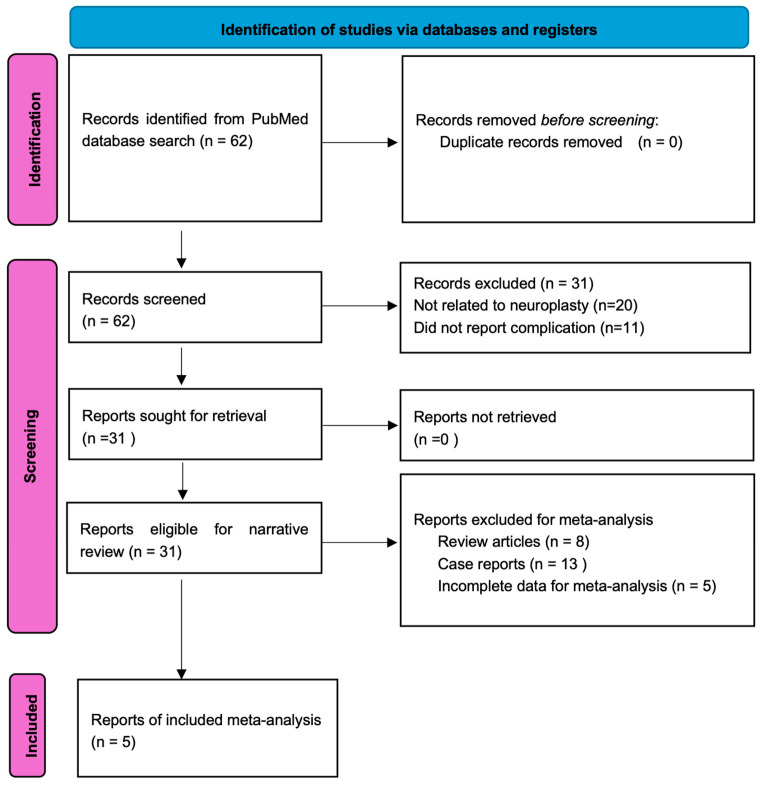
PRISMA flow diagram illustrating the study selection process for the narrative review and meta-analysis of neuroplasty-related complications.

Two independent reviewers screened titles, abstracts, and full texts. Disagreements were resolved through discussion or consultation with a third reviewer. The PRISMA flow diagram (Figure 1) summarizes the selection process.

### 2.2. Data Extraction and Quality Assessment

Data were extracted using a predefined template including study design, patient population, sample size, PEN technique, type and number of complications, and follow-up duration.

Study quality was assessed using the Newcastle–Ottawa Scale (NOS) for non-randomized studies, focusing on selection, comparability, and outcome domains.

### 2.3. Outcomes and Statistical Analysis

The primary outcome was the overall complication rate associated with PEN. Secondary outcomes included complication subtypes, severity (minor/moderate/severe), and timing.

For the quantitative synthesis, we performed a meta-analysis using a random-effects model (DerSimonian–Laird method) due to expected heterogeneity across studies.

Continuity corrections were applied when zero events were reported. Heterogeneity was assessed using the I^2^ statistic, with >75% indicating high heterogeneity. Publication bias was not formally assessed due to the limited number of included studies.

## 3. Results

### 3.1. Study Selection

A total of 62 articles were initially identified through a PubMed search using targeted keywords related to neuroplasty and its associated complications. After screening for relevance and eligibility, 31 studies were selected for inclusion in this narrative review (Figure 1). Of these, five observational studies that clearly reported both the number of treated patients and the number of complications were included in a quantitative analysis to estimate complication incidence. Inclusion criteria for the pooled analysis are detailed in the following section (Table 1).

All five cohort studies were independently assessed for risk of bias using the Newcastle–Ottawa Scale (NOS). Three studies (Sim et al., 2022 [22], Ceylan et al., 2019 [20], Ege et al., 2024 [17]) achieved NOS scores of 8 / 9, denoting high quality, while two studies (Talu et al., 2003 [13], Choi et al., 2017 [7]) scored 6 / 9, indicating moderate quality (Table 2). These findings confirm that the overall evidence base was of moderate to high quality.

In addition, 13 case reports and selected review articles were retained to explore the diversity of potential complications and to illustrate clinical scenarios that may benefit from structured post-procedural monitoring (Table 3). The remaining 13 studies included original articles and review articles providing qualitative information on complication types, severity, and contributing factors. This selective inclusion allowed for a balanced approach that synthesized the clinical experiences and insights reported in the existing literature through a narrative review while also enabling a focused meta-analysis of studies that met predefined criteria for quantitative evaluation.

### 3.2. Pathophysiological Basis of Epidural Neuroplasty: Implications for Complication Risk

Epidural adhesion or fibrosis, a primary target of percutaneous epidural neuroplasty, commonly results from inflammation, surgical trauma, disc herniation, or chronic degenerative spinal disorders [15,16,17]. Fibrotic tissue in the epidural space can tether spinal nerve roots, restricting their normal movement and causing chronic irritation and neuropathic pain [18]. Additionally, inflammatory mediators released from these fibrous tissues aggravate inflammation, promote nerve sensitization, and intensify pain.

The procedure of epidural neuroplasty (or epidural adhesiolysis) begins with catheter insertion into the epidural space, typically through the sacral hiatus (caudal approach) or via an interlaminar space, guided by fluoroscopy or epiduroscopy. After epidural access, contrast media is injected to delineate the extent of epidural fibrosis or adhesion, neural compression, and anatomical structures. Subsequently, mechanical manipulation using specially designed steerable catheters or balloon devices physically disrupts the fibrotic adhesions surrounding the nerve roots [1,4,5,17].

Pharmacologically, PEN utilizes corticosteroids to reduce inflammation, local anesthetics such as lidocaine or ropivacaine to provide analgesia, and hyaluronidase to facilitate the dispersion of injected medications and prevent re-adhesion formation [6,19,20]. Hypertonic saline and ozone have been employed to reduce edema and fibrosis, but there are potential neurotoxic risks, particularly if inadvertently administered intrathecally or intravascularly [8,21,22].

Given the complexity of spinal anatomy and the variability in the severity of epidural fibrosis, percutaneous epidural neuroplasty (PEN) carries inherent procedural risks. Anatomical factors—such as altered spinal architecture due to prior surgery or severe degenerative changes—can significantly complicate catheter navigation, thereby increasing the risk of complications, including dural disruption, cerebrospinal fluid (CSF) leakage, inadvertent intrathecal catheter placement, and direct nerve injury. Moreover, the pharmacological agents used in the procedure may cause neurotoxicity if improperly administered, emphasizing the critical need for precision in both catheter placement and drug delivery. Recognizing these pathophysiological mechanisms and procedural complexities underscores the necessity for meticulous patient selection, precise technique, and vigilant post-procedural monitoring to effectively reduce the risk of complications [12,13,17].

### 3.3. Complication Spectrum and Classification

Complications associated with percutaneous epidural neuroplasty can be broadly classified into four main categories: mechanical, neurological, infectious, and systemic. Each category comprises specific adverse events that vary widely in severity and clinical implications.

Mechanical Complications: These include dural puncture, catheter breakage, misplacement, or migration. Dural punctures can result in cerebrospinal fluid leaks, leading to post-dural puncture headaches and potential long-term morbidity if untreated. Catheter-related issues, such as breakage or retention, necessitate additional interventions, including surgical removal.

Neurological Complications: Neurological complications range from minor transient symptoms, like temporary numbness or mild paresthesia, to severe and potentially permanent neurological deficits, such as paraplegia due to epidural hematoma or direct nerve injury. Severe events are typically associated with prolonged recovery and significant impairment of quality of life.

Infectious Complications: Infection risks include superficial entry-site infections, deeper epidural abscesses, or even meningitis. These complications often present with systemic signs, such as fever, headache, neck stiffness, or neurological deficits, requiring prompt diagnosis and aggressive treatment to prevent lasting damage.

Systemic Complications: Systemic adverse events, although less common, can be life-threatening. These include cardiopulmonary arrest, reverse Takotsubo cardiomyopathy, severe allergic reactions, and pneumocephalus. These events typically arise from inadvertent intravascular or intrathecal injection of medications or complications related to high-pressure injection or catheter misplacement.

The classification of complications by severity (minor, moderate, and severe) provides valuable clinical insights into the need for structured observation and intervention. Minor complications generally resolve spontaneously or with minimal intervention, moderate complications require targeted treatment but usually without lasting sequelae, and severe complications necessitate immediate and aggressive intervention to prevent permanent disability or death.

Recognizing this spectrum of complications and their severity underscores the critical importance of post-procedural monitoring protocols. Early detection and rapid intervention can significantly influence patient outcomes, mitigating long-term complications and improving patient safety.

### 3.4. Complication Incidence and Pooled Analysis

To estimate the incidence of complications associated with PEN, we conducted a meta-analysis of five observational studies that clearly reported both the number of treated patients and the number of complications. Inclusion criteria were (1) clearly defined patient populations, (2) explicit reporting of complication counts and denominators, and (3) no overlap in patient datasets. Case reports and studies lacking denominator data as well as those reporting zero complications with small sample sizes were excluded from the quantitative synthesis to reduce statistical instability and bias. Table 1 summarizes key characteristics of the five observational studies included in the meta-analysis.

The five selected studies comprised a total of 1740 patients, among whom 120 complications were reported. Individual complication rates varied widely, ranging from 0.6% to 39.2%. Study-level complication rates and their 95% confidence intervals (CI) were calculated using the Wilson score method:

- Talu et al. (2003): 39.2% (95% CI: 33.6–45.1%) [13];
- Choi et al. (2017): 0.6% (95% CI: 0.2–1.6%) [7];
- Ceylan et al. (2019): 7.3% (95% CI: 3.4–15.0%) [20];
- Sim et al. (2022): 1.1% (95% CI: 1.1–2.1%) [22];
- Ege et al. (2024): 5.3% (95% CI: 2.1–12.4%) [17].

**Figure 2:**
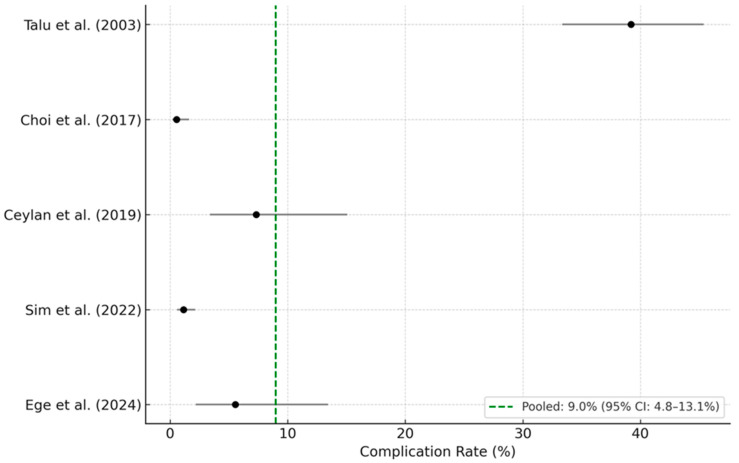
Forest plot of PEN-related complication rates (5 studies) showing pooled complication rates from five observational studies. Horizontal bars represent 95% confidence intervals. Note the high heterogeneity (I^2^ = 97.5%) [7,13,17,20,22].

A continuity correction was applied to all studies for consistency, and a random-effects meta-analysis was performed. The resulting pooled complication rate was 9.0% (95% CI: 4.8–13.1%), with substantial heterogeneity (I^2^ = 97.5%), indicating considerable between-study variation. The corresponding forest plot is presented in Figure 2, showing the study-level estimates and the overall pooled rate with confidence intervals.

Despite the variability, these findings suggest that PEN carries a non-trivial complication risk, with clinically meaningful adverse events occurring in approximately 1 out of every 10 patients. This estimate, based on high-powered and methodologically robust studies, underscores the importance of systematic complication monitoring and transparent reporting. Standardized definitions of adverse events and consistent follow-up protocols are essential to ensure comparability and safety in both clinical practice and future research.

The pooled complication rate, based on a random-effects model with continuity correction, was 9.0% (95% CI: 4.8–13.1%). Horizontal bars represent 95% Wilson score confidence intervals. Despite robust methodology, the meta-analysis revealed substantial heterogeneity (I^2^ = 97.5%), which limits the generalizability of this estimate.

Several likely sources of heterogeneity were identified. First, procedural techniques varied considerably across studies—including balloon neuroplasty, Racz catheter use, and epiduroscopy-guided interventions. Second, definitions of complications lacked standardization; while some studies reported only major adverse events, others included minor or transient symptoms. Third, patient populations differed in baseline pathology (e.g., spinal stenosis vs. failed back surgery syndrome), age, and comorbidities.

These differences underscore the importance of interpreting the pooled rate with caution. The statistical synthesis was intended to offer a broad estimate of complication incidence, not to imply consistency across clinical settings. Future studies using standardized definitions and stratified complication reporting are warranted.

### 3.5. Complication Type, Severity, and Timing

Complications were categorized into five broad types: mechanical, neurological, infectious, systemic, and miscellaneous. Across the five cohort studies (Table 1) and 13 published case reports (Table 3), a total of 133 complications were identified. Mechanical and neurological complications were the most frequently reported. A detailed classification is presented below:

- Mechanical complications (n ≈ 48):
  - Dural puncture: 11;
  - Catheter misplacement (vein, paravertebral, intradural): 18;
  - Catheter breakage or blockage: 9;
  - Problem during withdrawal: 3;
  - Intradural cyst formation: 3;
  - Procedural failure due to intrathecal passage: 3.
- Neurological complications (n ≈ 47):
  - Transient paresthesia: 19;
  - Prolonged paresthesia/numbness: 3;
  - Urinary/bowel dysfunction: 5;
  - Motor weakness: 6;
  - Paraplegia or irreversible deficit: 11;
  - Sexual dysfunction: 1;
  - Headache (post-dural or pneumocephalus-related): 2;
  - Persistent neurologic deficit: 5.
- Infectious complications (n ≈ 15):
  - Bacterial meningitis or ventriculitis: 3;
  - Epidural abscess: 3;
  - Local infections at catheter entry site: 7;
  - Systemic sepsis or delayed neurologic infection: 2.
- Systemic complications (n ≈ 13):
  - Hypotension or vasovagal reaction: 7;
  - Respiratory depression: 1;
  - Cardiopulmonary arrest or circulatory collapse: 2;
  - Pneumocephalus: 3.
- Miscellaneous or rare complications (n ≈ 10):
  - Imaging artifact mimicking subarachnoid hemorrhage: 1;
  - Persistent hiccup: 1;
  - Miscellaneous procedural anomalies (e.g., barotrauma, failed dye spread): 5.

Severity is categorized as follows:

- Mild (n ≈ 52): Headache, local discomfort, and transient symptoms.
- Moderate (n ≈ 38): Urinary retention, temporary motor weakness, and drug-related side effects.
- Severe (n ≈ 43): Epidural hematoma, spinal infection, persistent neurological deficit, and cardiovascular collapse.

Temporal patterns indicated the following:

- Immediate onset (0–1 h): Most pharmacologic or mechanical events (e.g., intrathecal injection, hypotension).
- Early onset (1–6 h): Epidural hematoma, pneumocephalus, respiratory issues.
- Delayed onset (6–48 h): Infectious complications and neurologic decline.

Identified risk factors included the following:

- Altered spinal anatomy (post-surgical, advanced stenosis or deformity).
- Use of high-risk agents (hypertonic saline, ozone).
- Elderly age or medical comorbidities.

These systematic findings underscore the necessity for structured monitoring protocols, particularly during the first 6 h following PEN. This window encompasses the majority of high-risk complications, offering a critical opportunity for early intervention to prevent irreversible harm. These data further reinforce our multidisciplinary recommendation for routine inpatient-level observation after PEN.

### 3.6. Clinical Case Highlights and Analysis of High-Risk Complications

The following section provides a qualitative synthesis of case reports. These individual reports are not included in the quantitative meta-analysis and are presented here solely to illustrate the diversity and severity of complications encountered in practice.

Severe complications, although infrequent, significantly impact patient outcomes and healthcare utilization. Analyzing specific clinical cases provides critical insights into the nature and implications of these high-risk complications.

Epidural Hematoma: A reported case involved an 81-year-old woman with dementia who developed bilateral leg paralysis five days post-procedure despite surgical intervention. Epidural hematoma, though rare, is particularly devastating and emphasizes the necessity for prompt recognition and emergency decompression [23].

Intracranial Hemorrhage: Another significant complication involved a 53-year-old patient experiencing persistent headaches and subsequently diagnosed with bilateral chronic subdural hematoma [24]. Another patient, a 42-year-old male, displayed signs resembling subarachnoid hemorrhage (SAH) post-procedure due to intrathecal contrast injection, which resolved spontaneously. These cases underscore the critical importance of cautious contrast administration and vigilant neurological monitoring [25].

Meningitis and Epidural Abscess: Cases such as a 69-year-old man developing bacterial meningitis following unsuccessful cervical PEN [26] and a 48-year-old diabetic male who suffered from MRSA-related epidural abscess and subsequent meningitis illustrate severe infectious risks [27]. Prompt recognition and aggressive antimicrobial management are crucial to patient recovery.

Spinal Cord and Nerve Injury: A noteworthy case involved a 19-year-old female who developed acute motor weakness in the opposite leg immediately after lumbar PEN, attributable to barotrauma-induced nerve root edema. This highlights the risks associated with high-pressure fluid administration and emphasizes careful procedural techniques [28].

Intradural Cyst Formation: A 65-year-old woman developed significant intradural cyst formation post-epiduroscopy, compressing the cauda equina, necessitating surgical intervention. This complication illustrates potential severe consequences of inadvertent dural injury [29].

Reverse Takotsubo Cardiomyopathy: A rare but life-threatening complication involved a 34-year-old woman who experienced profound cardiovascular compromise due to inadvertent intrathecal drug administration, underscoring the necessity for meticulous procedural vigilance and robust post-procedural cardiovascular monitoring [30].

Catheter Breakage and Retention: Several cases, including a 68-year-old man and a 41-year-old woman, involved catheter fragmentation requiring surgical retrieval [31,32]. This emphasizes the importance of device integrity, procedural technique precision, and prompt postoperative imaging.

These clinical case highlights demonstrate the broad and severe spectrum of potential complications associated with PEN. They also reinforce the critical importance of immediate and structured inpatient monitoring post-procedure to rapidly identify and manage these complications, thereby optimizing patient safety and outcomes.

## 4. Discussion

This study combines both a narrative and a systematic review to provide a comprehensive assessment of complications following percutaneous epidural neuroplasty (PEN). Through meta-analysis of five observational studies, we found a pooled complication rate of 9.0%, which is comparable to previously reported estimates in the literature despite substantial heterogeneity. Importantly, the narrative component enriches this quantitative finding by detailing a wider spectrum of adverse events—including rare but severe cases such as epidural hematoma, meningitis, and cardiopulmonary arrest—that often emerge within 6 h post-procedure. Compared with earlier studies focusing solely on procedural efficacy or limited safety profiles, our findings emphasize the dual need for quantitative surveillance and clinical contextualization. Taken together, the evidence supports both the clinical effectiveness of PEN in select patient populations and the critical importance of structured monitoring protocols to mitigate its potential risks.

While our analysis benefits from combining systematic and narrative evidence, several important limitations must be acknowledged. First, the small number of high-quality observational studies eligible for meta-analysis reflects the limited evidence base in this area. Many studies were excluded due to insufficient complication reporting, small sample sizes, or lack of clear denominators—factors that introduce potential selection and reporting bias. Furthermore, the absence of randomized controlled trials limits the ability to draw causal inferences about PEN-related risks. Given the limited number of included studies and substantial heterogeneity, formal GRADE assessment was not performed. However, study quality was assessed using the Newcastle–Ottawa Scale, and overall evidence strength was judged as moderate to low. In addition, the absence of a pre-registered protocol may introduce a risk of reporting bias, though all inclusion/exclusion criteria were transparently applied and described.

Second, while the included studies varied in patient demographics and procedural methods (e.g., balloon vs. Racz catheter techniques), inclusion was based on a shared core intervention—percutaneous epidural neuroplasty aimed at addressing spinal pain via epidural adhesion disruption. Despite these differences, all studies reported complications directly attributable to PEN, justifying their inclusion for pooled safety estimation.

Third, although the narrative synthesis provides valuable clinical insight through detailed case reports, these sources inherently lack generalizability and are subject to publication bias. Rare but serious complications tend to be overrepresented, while common but less severe outcomes may be underreported. As such, the case series serve primarily to illustrate the breadth and severity of potential risks rather than to define their frequency.

Taken together, these limitations highlight the urgent need for high-quality, prospective, multicenter studies employing standardized definitions of adverse events and robust reporting protocols. Future research should aim to stratify complication risk by patient characteristics (e.g., age, spinal pathology, surgical history), procedural techniques, and pharmacologic agents used. A centralized complication registry or post-procedural surveillance system could improve transparency, facilitate risk prediction, and ultimately guide best practices for procedural safety and post-procedural monitoring in interventional spine care.

Importantly, while the current evidence base remains limited, a consistent signal across both systematic and narrative data supports the need for vigilant clinical protocols following PEN—particularly in the immediate post-procedural period. The following section outlines the physiological and clinical rationale for structured inpatient monitoring and provides guidance for its integration into routine practice.

### 4.1. Medical Rationale for Structured Post-Procedural Inpatient Monitoring

Structured inpatient monitoring following PEN procedures is essential because of the temporal characteristics and potential severity of complications. Clinical evidence indicates that many serious complications, such as epidural hematoma, neurological deficits, and severe systemic reactions, typically manifest within the initial 6 h post-procedure [12,13,23,24,33,34]. Immediate post-procedural inpatient monitoring allows for healthcare providers to quickly identify early signs of complications, facilitating timely interventions to prevent irreversible damage.

Prompt identification and management of complications, such as epidural hematoma and intrathecal injection, significantly impact patient outcomes. The literature consistently highlights improved neurological outcomes when interventions, like hematoma evacuation, occur within the first 12 h following symptom onset [35]. Conversely, delayed recognition and intervention can lead to permanent neurological impairment or even death.

While most serious complications occur within 6 h, delayed-onset complications, including mild infections, can occur up to 14 days after PEN; a follow-up visit is recommended following acute-phase inpatient monitoring and discharge, particularly for high-risk patients.

Moreover, structured inpatient monitoring permits careful observation of pharmacological responses, especially in vulnerable populations, such as elderly patients or those with significant comorbidities [12,13]. Adverse reactions to medications administered during PEN, including local anesthetics, corticosteroids, or sedatives, can cause severe systemic effects, like respiratory depression, hypotension, or allergic reactions. Inpatient settings allow for immediate response to these events, significantly enhancing patient safety.

From a multidisciplinary perspective, structured inpatient monitoring facilitates comprehensive patient management through collaboration among anesthesiology, neurology, and rehabilitation specialists. This collaborative approach enables early rehabilitation interventions and management of transient neurological deficits and ensures patients are stable enough for discharge.

Additionally, structured monitoring aligns with best practices from similar procedures such as epidural anesthesia and lumbar puncture, both of which incorporate routine short-term inpatient observation to monitor for potential complications [34,35,36].

This standard of care reflects broader clinical consensus and emphasizes the ethical responsibility of healthcare providers to minimize patient risks through vigilant post-procedural care. While structured inpatient monitoring is clinically prudent—especially during the initial 6 h window—its universal application may not be feasible in all healthcare settings. Future cost-effectiveness studies and risk stratification (e.g., by age, comorbidities, or procedural complexity) are needed to guide resource-conscious implementation. Ethical frameworks must also balance patient safety with autonomy and system-level feasibility.

In summary, structured post-procedural inpatient monitoring following PEN is justified by the timing, severity, and clinical implications of potential complications. This practice is essential to optimize patient safety, enhance outcomes, and uphold high standards of care.

### 4.2. Recommendations for Clinical Practice and Future Directions

Based on the synthesis of clinical evidence, several recommendations emerge to enhance patient safety and procedural outcomes in PEN:

1. Patient Selection and Risk Stratification: Comprehensive pre-procedural assessments, including detailed medical history, imaging reviews, and consideration of patient-specific risk factors such as age, anatomical variations, previous spinal surgeries, and comorbid conditions should be strongly recommended. High-risk patients warrant heightened vigilance and possibly extended inpatient monitoring.
2. Procedural Best Practices: Adoption of meticulous procedural techniques is crucial to minimize mechanical complications. Practitioners should be rigorously trained in catheter manipulation, medication injection protocols, and the use of imaging modalities such as fluoroscopy or epiduroscopy. Avoidance or cautious use of potentially neurotoxic agents, such as hypertonic saline and ozone, is recommended.
3. Structured Inpatient Monitoring: This period should include regular neurological evaluations, monitoring for signs of infection and systemic reactions, and careful assessment of medication-related side effects. Because delayed-onset complications—including infections and neurologic deterioration—may occur up to 14 days after PEN, a follow-up visit is advised following discharge, particularly for high-risk patients.
4. Multidisciplinary Collaboration: Implementing a multidisciplinary team approach, involving anesthesiologists, neurologists, rehabilitation specialists, spine surgeons, and nursing staffs, can enhance patient management and early identification of complications. This collaborative framework ensures comprehensive care that addresses both immediate and long-term patient needs.
5. Patient Education and Informed Consent: Providing patients with clear information about potential complications, symptoms to watch for post-procedure, and detailed instructions regarding follow-up care can significantly enhance patient compliance and early complication detection post-discharge.
6. Future Research Directions: Prospective, multicenter studies and standardized complication-reporting registries are needed to further quantify complication rates, define high-risk patient populations, and refine procedural guidelines. Further studies should also evaluate the cost-effectiveness of structured inpatient monitoring in both high- and low-risk populations as well as the long-term clinical benefits of early complication detection.
7. Implementing these recommendations into clinical practice can significantly mitigate the risks associated with PEN, improving patient safety, procedural outcomes, and overall healthcare quality.

## 5. Conclusions

Percutaneous epidural neuroplasty (PEN) offers substantial therapeutic benefits for patients with chronic spinal pain syndromes; however, it is associated with a notable risk of complications ranging from minor transient symptoms to severe, life-threatening events. Analysis of clinical evidence underscores the critical necessity of structured short-term inpatient monitoring following PEN procedures, particularly within the initial 6 h post-procedure when severe complications are most likely to manifest. By adopting evidence-based patient selection criteria, meticulous procedural techniques, and multidisciplinary collaborative approaches, clinicians can significantly mitigate these risks. Continued research and standardized complication-reporting mechanisms are recommended to further refine safety guidelines and enhance patient outcomes. Ultimately, prioritizing patient safety through vigilant monitoring and proactive complication management is critical to optimizing outcomes. This represents both an ethical and clinical imperative in interventional spinal pain management.

## Figures and Tables

**Table 1:** Characteristics of the five selected studies included in the meta-analysis of PEN-related complications.

Study	Year	Country	Population and Indication	Sample Size (N)	Complications (n)	Reported Complications
Sim et al. [22].	2022	South Korea	LSS + RNR undergoing balloon PEN	793	9	Hypotension, paresthesia, local infection
Ceylan et al. [20]	2019	Turkey	FBSS treated with epiduroscopy	82	6	Dural puncture, mild infections, transient symptoms
Ege et al. [17]	2024	Turkey	Lumbar epidural fibrosis (post-/non-op)	72	4	Neurologic symptoms, hypotension
Talu et al. [13]	2003	Turkey	Mixed spinal pain; Racz neuroplasty	250	98	Dural puncture, catheter issues, infections
Choi et al. [7]	2017	South Korea	LSS patients with/without sarcopenia treated with balloon PEN	543	3	Mild hypotension, transient discomfort

**Table 2:** Newcastle–Ottawa Scale Scores.

Authore (Year)	Selection	Comparability	Outcome	Total
Sim et al. (2022) [22]	4	2	2	8
Ceylan et al. (2019) [20]	4	2	2	8
Ege et al. (2024) [17]	4	2	2	8
Talu et al. (2003) [13]	4	0	2	6
Choi et al. (2017) [7]	4	0	2	6

**Table 3:** Summary of case reports describing complications following PEN.

First Author	Year	Country	Complication Type	Description
Kim, CH et al. [23]	2023	Korea	Hematoma/Neurologic	Massive epidural hematoma → permanent paralysis
Kim, SB et al. [24]	2014	Korea	Neurologic	Bilateral subdural hematoma
Oh, CH et al. [25]	2013	Korea	Imaging artifact	Contrast mimicking SAH on imaging
Noh, SM et al. [26]	2020	Korea	Infectious	Bacterial meningitis and ventriculitis
Lee, HY et al. [27]	2015	Korea	Infectious/Neurologic	Epidural abscess → cerebellar infarction
Lim, YS et al. [28]	2015	Korea	Mechanical	Barotrauma-induced acute motor weakness
Ryu, KS et al. [29]	2012	Korea	Structural	Iatrogenic intradural cyst formation
Lee, CH et al. [30]	2015	Korea	Systemic	Reverse Takotsubo cardiomyopathy post-intrathecal injection
Kim, TH et al. [31]	2016	England	Mechanical	Catheter breakage during procedure
Kang JH et al. [32]	2015	Korea	Mechanical	Retained Racz catheter fragment
Ho, KY et al. [33]	2008	USA	Neurologic	Acute monoplegia after adhesiolysis
Beyaz, SG et al. [8]	2018	India	Systemic	Cardiopulmonary arrest + pneumocephalus post-O_2_–O_3_
Torman, H et al. [9]	2017	Turkey	Systemic	Severe headache and pneumocephalus

## Data Availability

The datasets generated during and/or analyzed during the current study are available from the corresponding author on reasonable request.
